# Flower nectar trichome structure of carnivorous plants from the genus butterworts *Pinguicula* L. (Lentibulariaceae)

**DOI:** 10.1007/s00709-019-01433-8

**Published:** 2019-08-19

**Authors:** Krzysztof Lustofin, Piotr Świątek, Vitor F. O. Miranda, Bartosz J. Płachno

**Affiliations:** 1 Department of Plant Cytology and Embryology, Institute of Botany, Faculty of Biology, Jagiellonian University in Kraków, 9 Gronostajowa St, 30-387 Kraków, Poland; 2 Department of Animal Histology and Embryology, University of Silesia in Katowice, 9 Bankowa St, 40-007 Katowice, Poland; 3 Departamento de Biologia Aplicada à Agropecuária, Universidade Estadual Paulista (Unesp), Faculdade de Ciências Agrárias e Veterinárias, Jaboticabal, São Paulo, Brazil

**Keywords:** Butterworts, Carnivorous plant, Floral micromorphology, Lentibulariaceae, Nectary structure, *Pinguicula*, Spur, Trichomes

## Abstract

*Pinguicula* (Lentibulariaceae) is a genus comprising around 96 species of herbaceous, carnivorous plants, which are extremely diverse in flower size, colour and spur length and structure as well as pollination strategy. In *Pinguicula*, nectar is formed in the flower spur; however, there is a gap in the knowledge about the nectary trichome structure in this genus. Our aim was to compare the nectary trichome structure of various *Pinguicula* species in order to determine whether there are any differences among the species in this genus. The taxa that were sampled were *Pinguicula moctezumae*, *P. moranensis*, *P. rectifolia*, *P. emarginata* and *P. esseriana*. We used light microscopy, histochemistry, scanning and transmission electron microscopy to address those aims. We show a conservative nectary trichome structure and spur anatomy in various Mexican *Pinguicula* species. The gross structural similarities between the examined species were the spur anatomy, the occurrence of papillae, the architecture of the nectary trichomes and the ultrastructure characters of the trichome cells. However, there were some differences in the spur length, the size of spur trichomes, the occurrence of starch grains in the spur parenchyma and the occurrence of cell wall ingrowths in the terminal cells of the nectary trichomes. Similar nectary capitate trichomes, as are described here, were recorded in the spurs of species from other Lentibulariaceae genera. There are many ultrastructural similarities between the cells of nectary trichomes in *Pinguicula* and *Utricularia*.

**Electronic supplementary material:**

The online version of this article (10.1007/s00709-019-01433-8) contains supplementary material, which is available to authorized users.

## Introduction

Lentibulariaceae L. is a monophyletic family within the Lamiales (APG IV 2016; Schäferhoff et al. 2010) and contains about 360 species. This family consists of three genera of carnivorous plants: *Pinguicula* L., *Genlisea* A. St.-Hil. and *Utricularia* L. (e.g. Juniper et al. 1989; Jobson et al. 2003; Müller et al. 2004; Fleischmann and Roccia 2018). The family Lentibulariaceae probably originated about 42 million years ago (Ibarra-Laclette et al. 2013; Silva et al. 2018). Most probably, the genus *Pinguicula* originated in South America. It is the second largest genus of this family and contains about 96 currently recognised species. *Pinguicula* species occur on all of the continents except for Australia, with its centre of diversity in Central America (Casper 1966; Beck et al. 2008; Roccia et al. 2016; Fleischmann and Roccia 2018).

Although all Lentibulariaceae genera contain plants that are carnivorous herbs, they use the different strategies for capturing their prey. The *Genlisea* species have subterranean eel (lobster-pot) traps for trapping small water/soil invertebrates, while the *Utricularia* species form suction bladders (e.g. Lloyd 1942; Juniper et al. 1989; Reut 1993; Płachno et al. 2007, 2019a; Poppinga et al. 2016). *Pinguicula* species are active ‘flypapers’ that have a basal rosette of slightly modified leaves for trapping small invertebrates. *Pinguicula* leaves have two types of epidermal glandular trichomes (stalked and sessile) on their upper surfaces. Stalked trichomes carry mucilaginous droplets for trapping prey, which the sessile trichomes produce digestive enzymes and absorb the nutrients from digested prey (e.g. Heslop-Harrison 1970; Heslop-Harrison and Heslop-Harrison 1980; Legendre 2000; Adlassnig et al. 2010). Some species, e.g. *P. gigantea* and *P. longifolia*, also have mucilage trichomes on their lower leaf surface (Fleischmann and Roccia 2018). Their natural prey is mainly small flying Diptera (Nematocera) and other insects (Coleoptera, Thysanoptera, Lepidoptera) as well as springtails (Collembola), mites, spiders and gastropods (e.g. Karlsson et al. 1987; Zamora 1990, 1999; Heslop-Harrison 2004; Adler and Malmqvist 2004; Alcalá and Domínguez 2003, 2005; Darnowski et al. 2018).

**Fig. 1:**
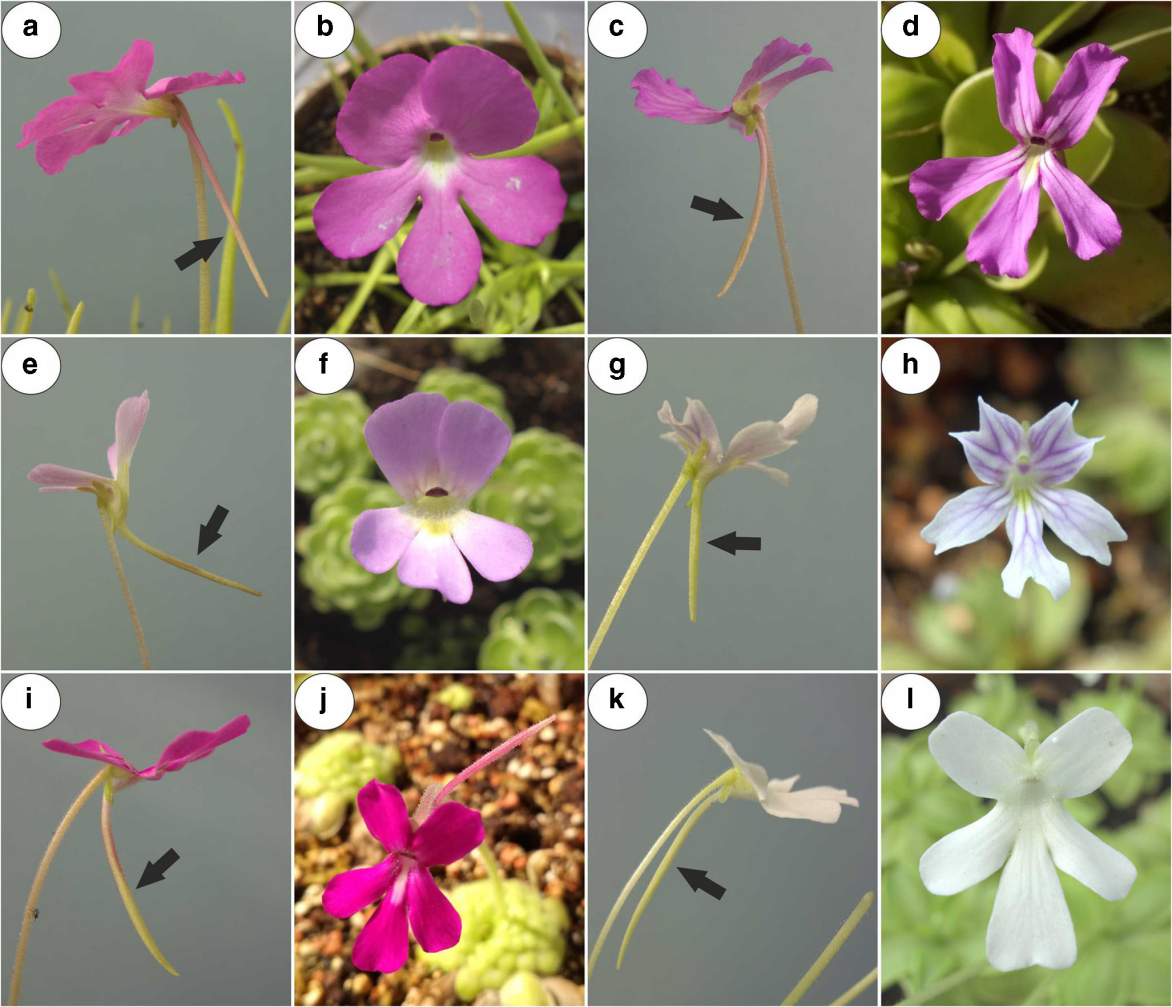
General floral morphology of the examined *Pinguicula* species; note the presence of spurs (arrow). **a**, **b***P. moctezumae*. **c**, **d***P. rectifolia*. **e**, **f***P. esseriana*. **g**, **h***P. emarginata*. **i**, **l***P. moranensis*

Members of Lentibulariaceae have zygomorphic flowers that have a sympetalous corolla that is bilobed. The upper lip is formed by two and the lower lip by three fused petals. In all three genera, there are flower spurs, which are tubular outgrowths of the perianth organs and contain nectar for their pollinators (Casper 1966; Taylor 1989; Fischer et al. 2004; Fleischmann et al. 2010; Aranguren et al. 2018; Płachno et al. 2017, 2018, 2019b, c). In *Pinguicula*, the corolla is throat-like and its shape is either distinctly zygomorphic (Fig. 1a–l) with the lower lip spreading widely from the upper lip or nearly isolobous and radial (Fleischmann and Roccia 2018). The flowers vary in their sizes, colours (from red, violet, pink, blue to white) and spur size and shape (Fig. 1a–l; see Casper 1966; Roccia et al. 2016; Lampard et al. 2016).

*Pinguicula* species are extremely diverse in their pollination strategies. Schnell (1976) proposed that the tubular *Pinguicula* flowers are pollinated by long-tongued insects. Hymenoptera (bumblebees, carpenter bees, honeybees and other smaller bees) are the primary flower visitors in the *Pinguicula* species in the south-eastern USA: *Pinguicula ionantha*, *P. lutea* and *P. planifolia* (Molano-Flores et al. 2018). The flowers of the Mediterranean *P. vallisneriifolia* have specialised floral traits (large, spurred flowers, contrasting colours, floral guides and the presence of nectar), which suggest a bee type of flower. Although *P. vallisneriifolia* flowers are visited by hymenoptera, thrips (*Thripsmeridionalis*) and small beetles (*Eusphalerumscribae*) are the primary pollen vectors for this species (Zamora 1999). Thus, despite their specialised floral traits for bee pollination syndrome, the main pollinators belong to other groups (Zamora 1999). According to Heslop-Harrison (2004), the flowers of *P. vulgaris* are primarily bee pollinated. The flowers of *P. alpina* are adapted for insect pollination mainly by flies but also by bees (Molau 1993; Heslop-Harrison 2004). In Gotland, *P. alpina* flowers were visited by both flies and bees: *Melanostomascalare*, *Platycheirusalbimanus*, *Lasioglossumfratellum*, *Lasioglossumalbipes*, *Dasysyrphus* and members of Empididae and Anthomyiidae (Nordin 2015). According to this author, short-tongued insects can also pollinate this species because both the throat and spur in *P. alpina* flowers are wide, which enables penetration. The Mexican *Pinguicula* species (*P. moranensis*), which produce large, coloured, hercogamous flowers that have long spurs, are pollinated by Lepidoptera (Zamudio 2001; Villegas and Alcalá 2018, see Fig. 5 in Villegas and Alcalá 2018, which presents a beautiful “Schematic representation of pollination in *Pinguicula moranensis*”). According to Abrahamczyk et al. (2017), *Pinguicula macrophylla* and *P. moctezumae*are are also pollinated by Lepidoptera. *Pinguicula hemiepiphytica* and the related *Pinguicula laueana* have purple-red or red flowers that have long spurs and are visited by hummingbirds and, most probably, are pollinated by them (Lampard et al. 2016); however, this should be verified because no *Pinguicula* pollen grains have been observed on the bodies of hummingbirds.

Only a few species have been studied to determine the occurrence of nectar in *Pinguicula* flowers. Zamora (1999) noted that *P. vallisneriifolia* only produces traces of nectar. Abrahamczyk et al. (2017) showed that the nectar sugar composition varies in the species that are pollinated by different groups of insects, e.g. species that are pollinated by butterflies (*P. macrophylla* and *P. moctezumae*) had fructose-dominated nectar, while species that are classified as being pollinated by bees and wasps had sucrose-dominated nectar (*P. gigantea*) or hexose-dominated nectar (*P. leptoceras*). *P. alpina*, which was classified by Abrahamczyk et al. (2017) as fly-pollinated species, also had sucrose-dominated nectar. However, this species is also pollinated by bees (Molau 1993).

In *Utricularia*, the spur is treated as a nectary and nectar is produced by small glandular trichomes (Płachno et al. 2017, 2018, 2019b, c). The ultrastructure of these trichomes was analysed by Płachno et al. (2017, 2019c). Moreover, the small spur trichomes in *Genlisea* also produce nectar (Fleischmann 2012; Aranguren et al. 2018). However, it is important to note that there is a gap in our knowledge about the nectary trichome structure in *Pinguicula*. Such data will be very useful in the future in order to create an ancestral state reconstruction and phylogenetic hypothesis of the pollination syndrome in Lentibulariaceae. Therefore, our aim was to compare the nectary trichome structure of various *Pinguicula* species in order to determine whether there are any differences among species in the genus. In case of *P. moranensis*, we used a typical flowered plant as well as the white-flowered form to determine whether there are any differences between these forms.

## Material and methods

### Plant material

The plant material (*Pinguicula moctezumae* Zamudio & R. Z. Ortega, *P. moranensis* H. B. K., *P. rectifolia* Speta & Fuchs, *P. emarginata* Zamudio Ruiz & Rzedowski and *P. esseriana* B. Kirchner; Fig. 1a–l) was bought from the Best Carnivorous Plants Store (KamilPásek, Ostrava, Czech Republic). The plants were later cultivated in the Botanical Garden of the Jagiellonian University in Kraków. For each species, minimum ten flowers (at middle stage of the anthesis) from ten individual plants were collected and studied. All studied flowers were at the same, comparable stage. The length of the spurs of investigated species was *Pinguicula moctezumae* (25) 28–35 (38) mm; *P. moranensis* (18) 25–35 (44) mm; *P. rectifolia* about 30 mm; *P. emarginata* about 7 mm and *P. esseriana* (10) 15–20 (30) mm (Roccia et al. 2016; http://www.pinguicula.org/pages/pages_principales/content.html).

## Methods

The flower spurs were examined using light microscopy (LM), scanning electron microscopy (SEM) and transmission electron microscopy (TEM) as follows. Small fragments of the apical part of the spurs were fixed in a mixture of 2.5% or 5% glutaraldehyde with 2.5% formaldehyde in a 0.05-M cacodylate buffer (Sigma; pH 7.2) overnight or for several days, washed three times in a 0.1-M sodium cacodylate buffer and post-fixed in a 1% osmium tetroxide solution at room temperature for 1.5 h. Dehydration using a graded ethanol series, infiltration and embedding using an epoxy embedding medium kit (Fluka) followed. After polymerisation at 60 °C, sections for the TEM were cut at 70 nm using a Leica Ultracut UCT ultramicrotome, stained with uranyl acetate and lead citrate (Reynolds 1963) and examined using a Hitachi H500 transmission electron microscope (Hitachi, Tokyo, Japan), which is housed in the University of Silesia in Katowice, at an accelerating voltage of 75 kV. The semi-thin sections (0.9–1.0 μm thick) that were prepared for the LM were stained with aqueous methylene blue/azure II (MB/AII) for 1–2 min (Humphrey and Pittman 1974) and examined using an Olympus BX60 light microscope for the general histology. The periodic acid-Schiff (PAS) reaction for the LM (semi-thin sections) was also used to reveal the presence of insoluble polysaccharides and Sudan Black B was used to detect the presence of lipids and cuticle material (Jensen 1962).

Additionally, material that was embedded in Technovit 7100 (Kulzer, Germany) was also observed. The material was fixed in a mixture of 2.5% or 5% glutaraldehyde with 2.5% formaldehyde in a 0.05-M cacodylate buffer (Sigma; pH 7.2) overnight, washed three times in a 0.1-M sodium cacodylate buffer, dehydrated in a graded ethanol series for 15 min at each concentration and kept overnight in absolute ethanol. Later, the samples were infiltrated for 1 h each in 3:1, 1:1 and 1:3 (v/v) mixtures of absolute ethanol and Technovit and then stored for 12 h in pure Technovit. The resin was polymerised by adding a hardener. The material was sectioned to 5 μm thick using a rotary microtome (Microm, AdamasInstrumenten), stained with 0.1% toluidine blue O (TBO) and mounted in Entellan synthetic resin (Merck).

Living, non-fixed spurs were cut using a razor blade and observed under UV light using a Nikon Eclipse E400 microscope to determine any autofluorescence of the cuticle. In order to identify the main classes of the chemical compounds that are present in the spur tissues, histochemical procedures with the spurs of fresh or fixed flowers using Sudan III, Sudan Black B and Lugol’s solution were performed to detect the total lipids, starch grains and proteins (Johansen 1940), respectively.

For the SEM, the spur traps were fixed (as above) and later dehydrated and critical point dried using CO_2_. They were then sputter-coated with gold and examined at an accelerating voltage of 20 kV using a Hitachi S-4700 scanning electron microscope, which is housed in the Institute of Geological Sciences, Jagiellonian University in Kraków, Poland.

### Statistical analysis

We measured the trichomes length and trichomes head diameter for each species (Table 1). The numbers of flowers used for measurements were three for *P. moranensis, P. moctezumae, P. rectifolia* and two for *P. esseriana, P. emarginata*. Each variable was tested using the Shapiro-Wilk W-test for normality. The homogeneity of variance was assessed with Levene’s test. Statistical differences in trichomes length, as well as trichomes head diameter between each *Pinguicula* species, were assessed using the Kruskal-Wallis nonparametric one-way ANOVA, followed by multiple comparison of average ranks for all trials test. Statistical analyses were performed on raw data using Statistic 13 software (StatSoft Inc.). Data from measurements of trichomes length and head diameter were expressed in μm as mean ± SD. Data were considered statistically significant at **p < 0.01 and ***p < 0.001.

**Table 1:** Measurements [average trichome length and head diameter (μm ± SD)] of the nectary *Pinguicula* trichomes (*n* = 20 for each species)

Species	*P. moctezumae*	*P. esseriana*	*P. emarginata*	*P. moranensis*	*P. rectifolia*
Trichome length (μm)	34.41 ± 3.94	45.36 ± 3.70	32.74 ± 3.14	42.41 ± 6.22	32.49 ± 2.31
Head diameter (μm)	26.96 ± 1.83	32.50 ± 1.74	22.42 ± 1.84	31.20 ± 2.74	24.84 ± 1.49

## Results

**Fig. 2:**
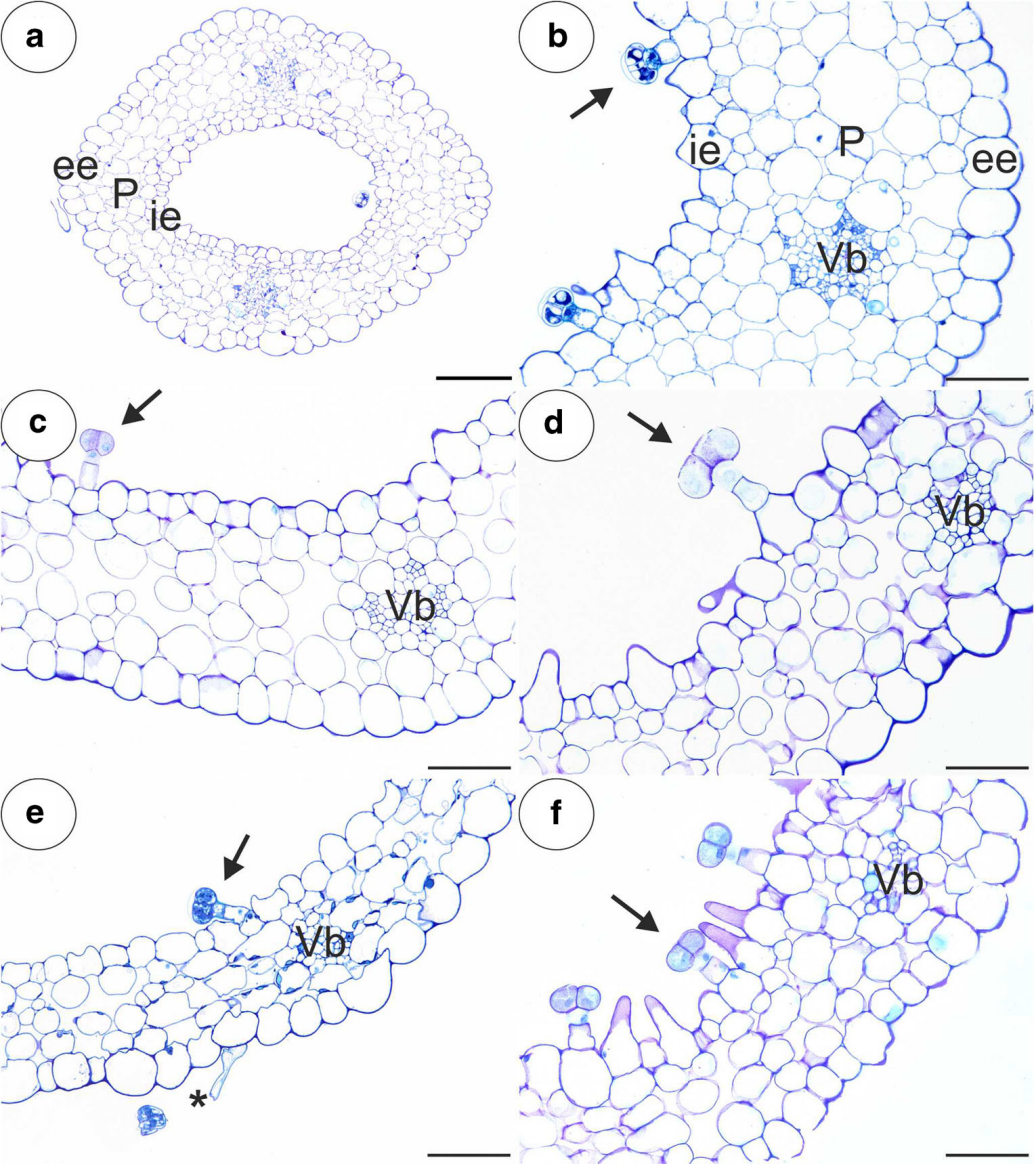
General anatomy of the examined *Pinguicula* spurs; Part of the sections through the spurs showing the inner epidermis (ie), external epidermis (ee), parenchyma (P), vascular bundles (Vb), outer glandular trichome (star) and nectary trichomes (arrow). **a**, **b***P. moctezumae*; scale bar respectively = 100 μm and 50 μm. **c***P. rectifolia*; scale bar = 50 μm. **d***P. esseriana*; scale bar = 50 μm. **e***P. emarginata*; scale bar = 50 μm. **f***P. moranensis*; scale bar = 50 μm

**Fig. 3:**
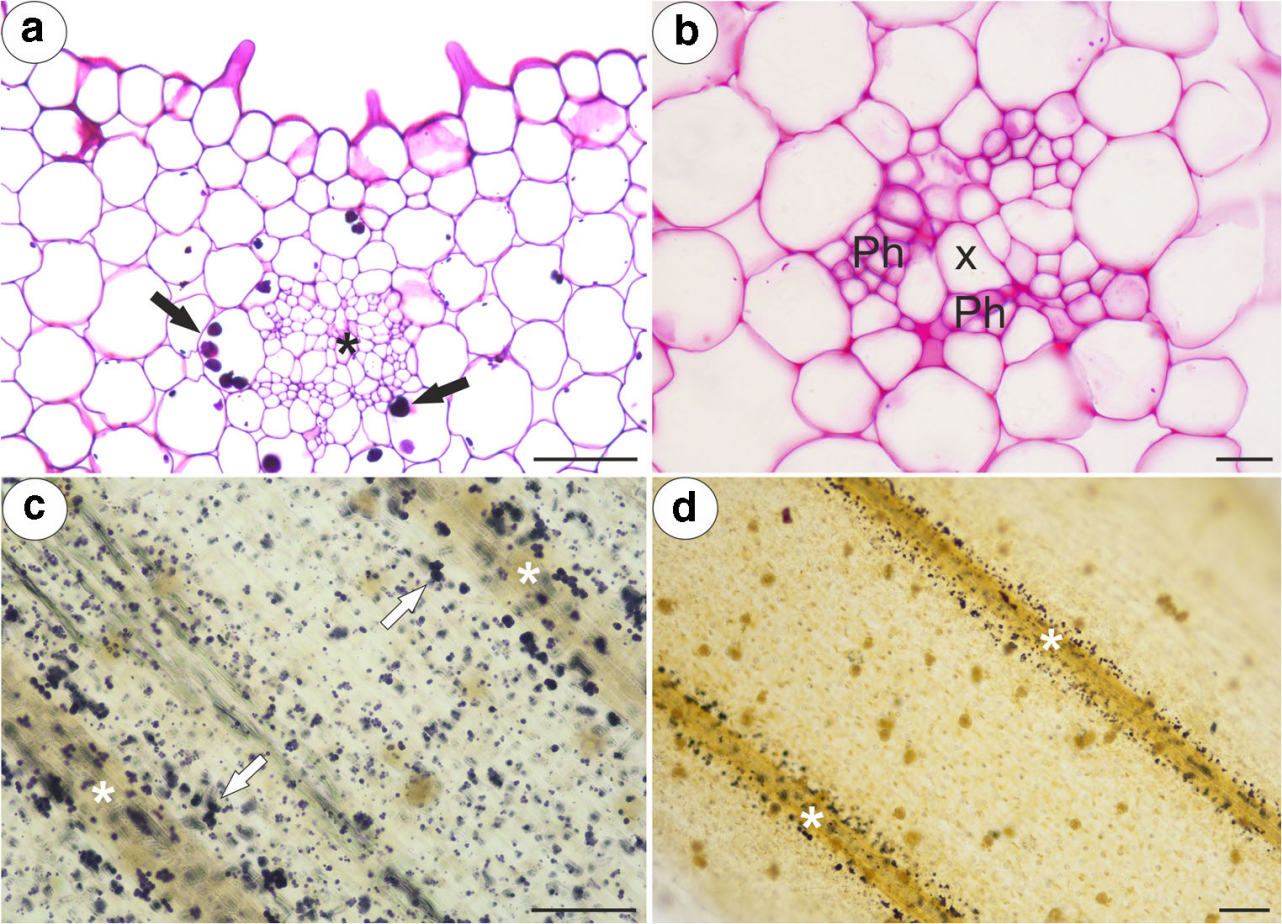
PAS reaction and Lugol’s staining of the examined *Pinguicula* spurs; note the presence of starch grains (arrow) around the vascular bundle (star), which contains the xylem (X) and phloem (Ph). **a***P. moctezumae*, scale bar = 50 μm. **b***P. moranensis*, scale bar = 10 μm. **c***P. moctezumae*, scale bar = 100 μm. **d***P. rectifolia*, scale bar = 100 μm

**Fig. 4:**
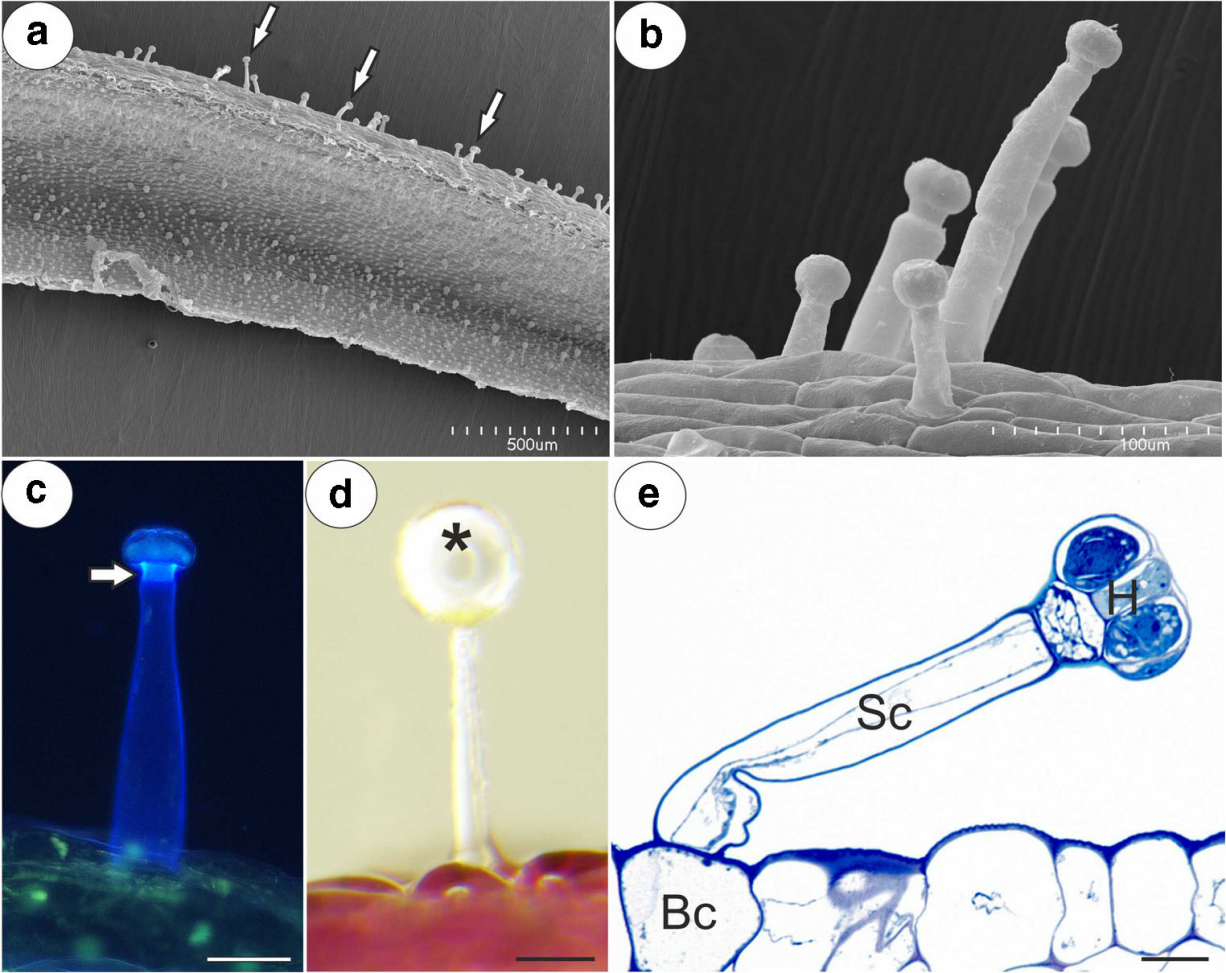
Micromorphology and anatomy of a glandular trichome located on the outer surface of a *P. moranensis* spur. **a**, **b** Micromorphology of the interior (S) and outer glandular trichomes of the spur (arrow); scale bar = 500 μm and 100 μm, respectively. **c** Autofluorescence of the outer trichome cell wall; note the bright fluorescence showing the cell wall of the pedestal cell that is heavily impregnated with cutin (arrowhead); scale bar = 50 μm. **d** General morphology of an outer trichome with a secretion droplet (star) on the top; scale bar = 50 μm. **e** Anatomy of an outer trichome showing the basal cell (Bc), stalk cell (Sc) and trichome head (H); scale bar = 10 μm

Any similar results for the five studied *Pinguicula* species were grouped and are presented together and the differences between them were highlighted in the text. We did not find the differences in the structure of flowers between typical and white-flowered forms of *P. moranensis*. The general anatomy of the spur was the same across the investigated species. In a transverse section, the wall of the spur was composed of several cell layers: the internal epidermis, layers of parenchyma cells and the outer epidermis (Fig. 2a–f). The parenchyma cells were non-glandular. There were from three to eight layers of parenchyma and the number of layers depended on the species (for example, three to four in *P. emarinata* and four to eight in *P. moctezumae*). The collateral vascular bundles, each of which contained both a xylem and phloem, occurred in the ground parenchyma (Fig. 2a–f, Fig. 3a, b). The parenchyma cells of *P. moctezumae*, *P. emarginata* and *P. rectifolia*, which surround the vascular bundles, contained amyloplasts with large starch grains (Fig. 3a, d). In *P. moctezumae*, starch grains also occurred in the other parenchyma cells (Fig. 3c). In *P. esseriana*, the occurrence of starch grains depended on the flowers. Both the external and internal epidermis of the spur had capitate glandular trichomes (Fig. 4a). In all of the examined species, there were long-stalked glandular trichomes at the external spur surface (Fig. 4b–e). Each of this type of trichome consisted of a single basal cell, stalk cells (up to four—*P. moranensis*, which mostly occurred in one or two cells), a pedestal cell and a multi-celled head (Fig. 4e). The number of head cells varied in the species (up to 16 cells). The pedestal cell was a barrier cell with lateral walls impregnated with cutin. The head cells were glandular and produced droplets of secretion (Fig. 4d).

**Fig. 5:**
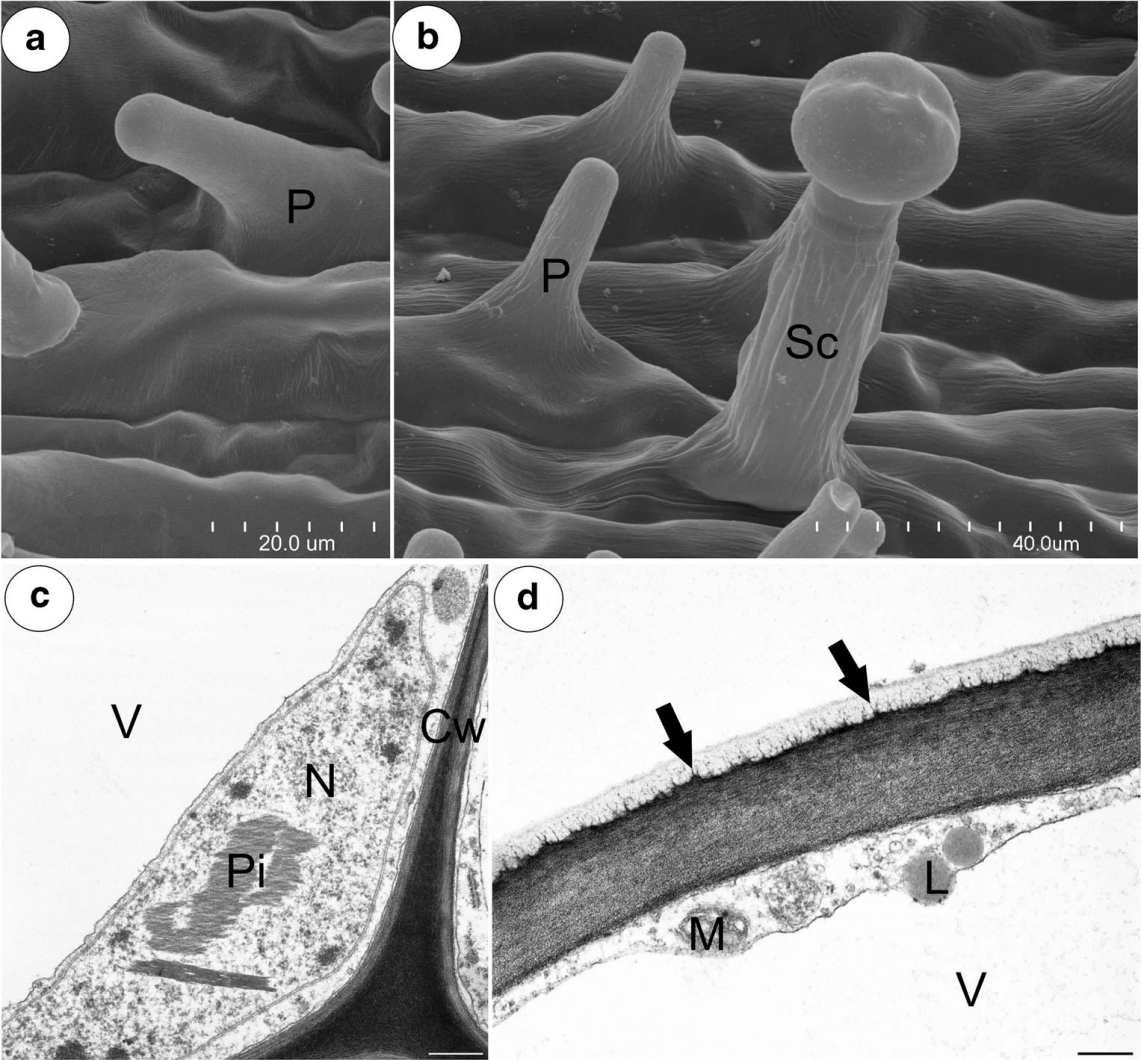
Structure of the *Pinguicula* papillae. **a**, **b** Micromorphology of *P. esseriana* and *P. moctezumae* papillae, respectively; note the papillae (P) with an almost smooth cuticular surface (**a**) and cuticular striations (**b**). These striations were also observed on cuticular surface of glandular trichome stalk cell (Sc); scale bar = 20 μm and 40 μm. **c**, **d**, respectively. Ultrastructure of *P. moctezumae* papillae; note the paracrystalline protein inclusion (Pi) in the nucleus (N), the cell wall (Cw) polysaccharide fibrillary network in the cuticle layer (arrow), lipid droplets (L) with mitochondria (M) in the cytoplasm and huge vacuoles (V); scale bar = 0.5 μm and 0.8 μm, respectively

**Fig. 6:**
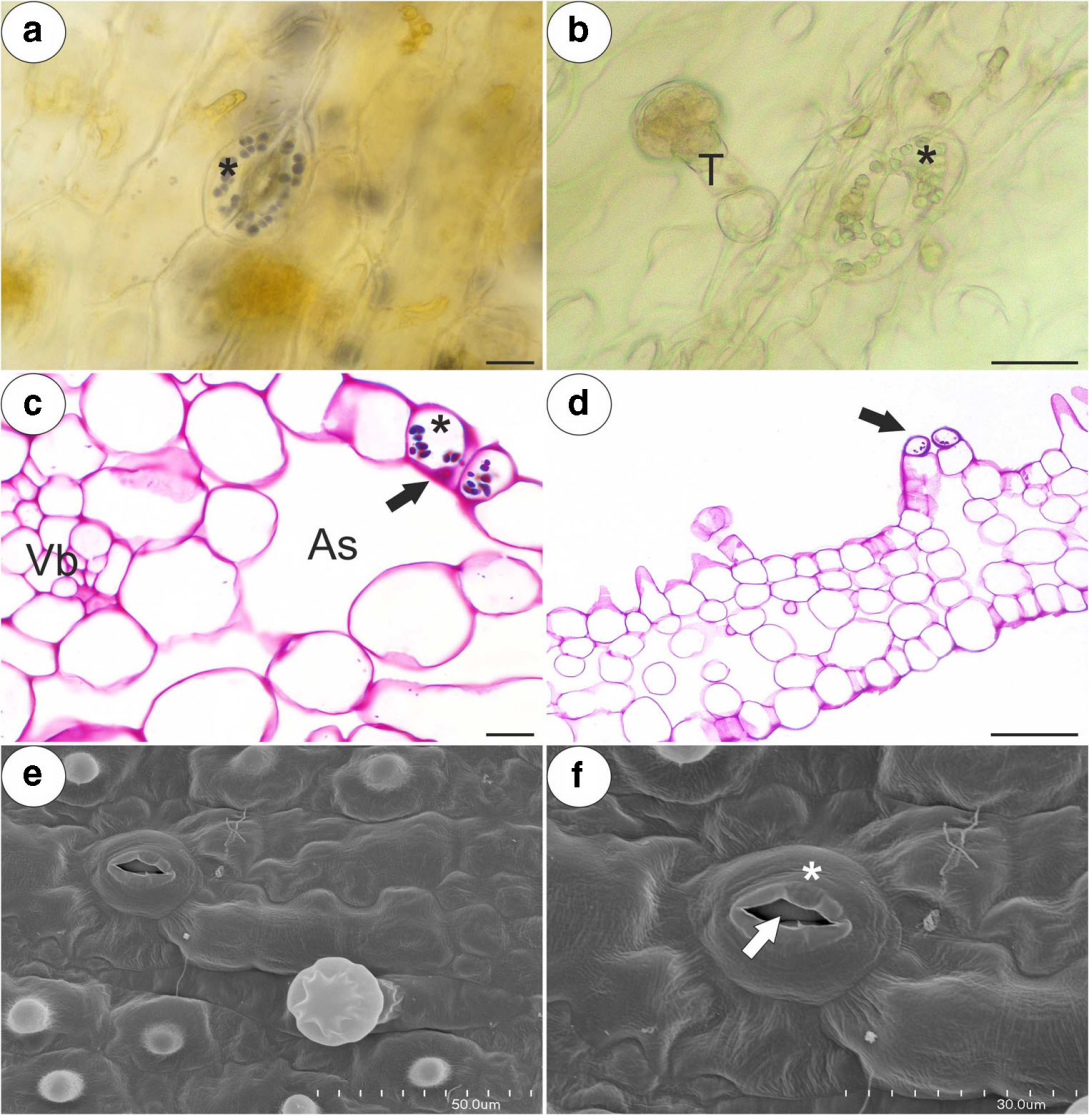
Morphology and anatomy of the examined *Pinguicula* stomata; note the guard cells (star) of the stoma with starch grains, the stoma pore (arrow), the air space below stoma (As) and a glandular trichome (T). **a**, **b** Morphology of *P. rectifolia* and *P. moranensis* stoma; scale bar = 10 μm and 25 μm, respectively. **c**, **d** PAS reaction of the *P. esseriana* and *P. moranensis* stoma; scale bar = 10 μm and 50 μm, respectively. **e**, **f** Micromorphology of the *P. rectifolia* stoma; scale bar = 50 μm and 30 μm, respectively

The inner epidermis formed short unicellular papillae that had an almost smooth surface (*P. esseriana*, Fig. 5a) or papillae with delicate striations (Fig. 5b). The papillae were highly vacuolated not glandular. The nuclei of the papillae had a paracrystalline protein inclusion (Fig. 5c). The papillae also had a thick cuticle. A polysaccharide fibrillary network of electron-dense ramification occurred in the cuticle layer (Fig. 5d). Stomata were observed in the inner epidermis of the spur (Fig. 6a–f). The stoma cells contained starch grains (Fig. 6a–d). Sometimes, the stomata were elevated above the surface of the epidermis (Fig. 6d).

**Fig. 7:**
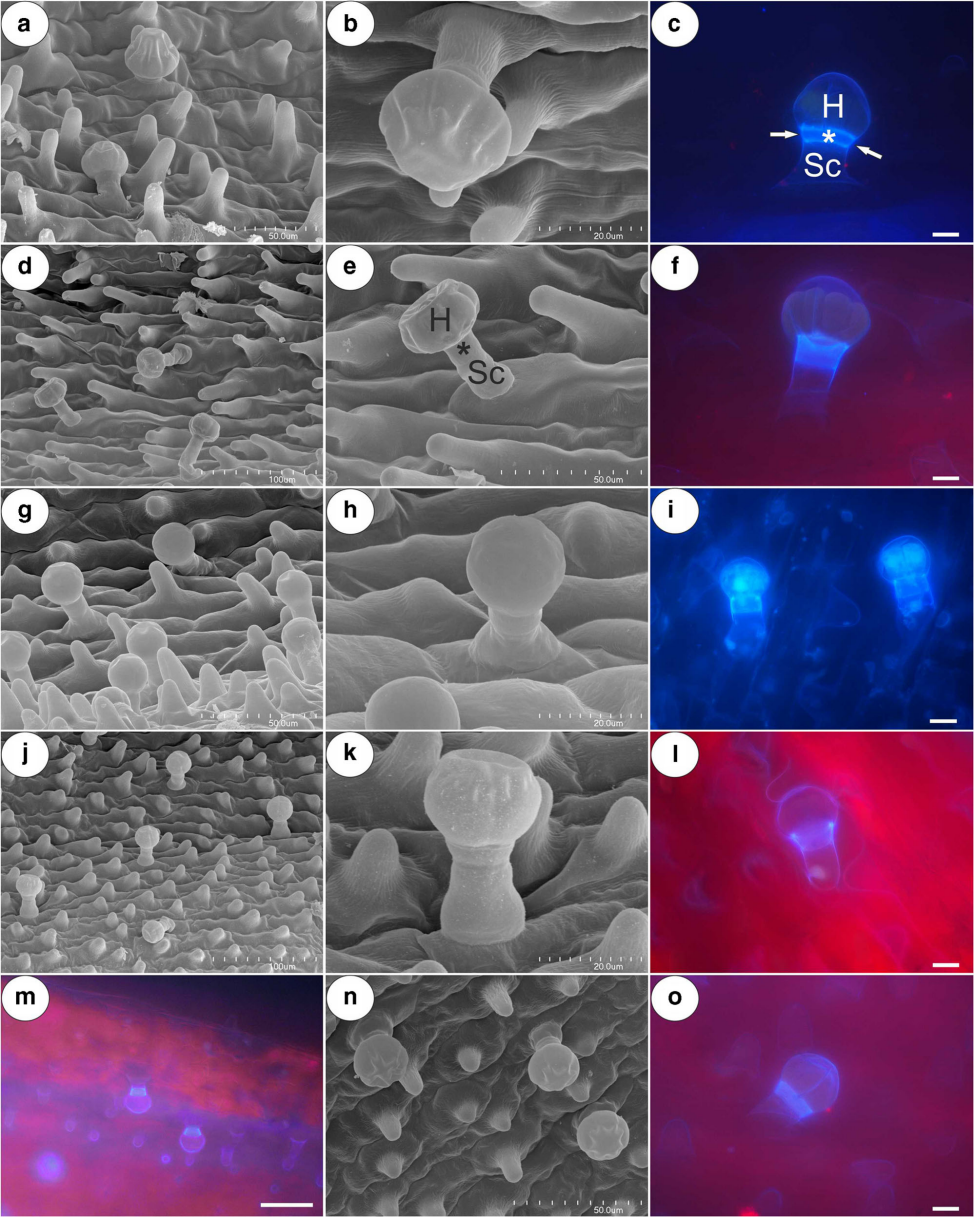
Micromorphology and autofluorescence of the glandular trichomes of the examined *Pinguicula* species; note the stalk cell (Sc), trichome head (H) and bright fluorescence showing the cell wall (arrow) of pedestal cell that is heavily impregnated with cutin (star). **a–c***P. moctezumae*, scale bar = 50 μm, 20 μm, 10 μm, respectively. **d–f***P. esseriana*, scale bar = 100 μm, 50 μm, 10 μm, respectively. **g–i***P. emarginata*, scale bar = 50 μm, 20 μm, 10 μm, respectively. **j–l***P. moranensis*, scale bar = 100 μm, 20 μm, 10 μm, respectively. **m–o***P. rectifolia*, scale bar = 50 μm, 50 μm, 10 μm, respectively

**Fig. 8:**
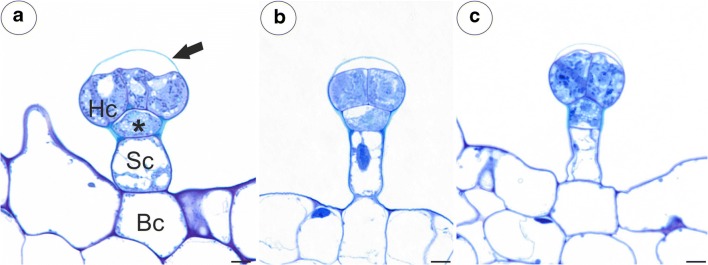
Anatomy of the glandular trichomes of the examined *Pinguicula* species. **a***P. moctezumae*; Part of a section through the glandular trichome showing the basal cell (Bc), stalk cell (Sc), pedestal cell (star), head cell (Hc) and cuticle separating from the cell wall (arrow); scale bar = 5 μm. **b***P. moranensis*; scale bar = 5 μm. **c***P. emarginata*; scale bar = 5 μm

**Fig. 9:**
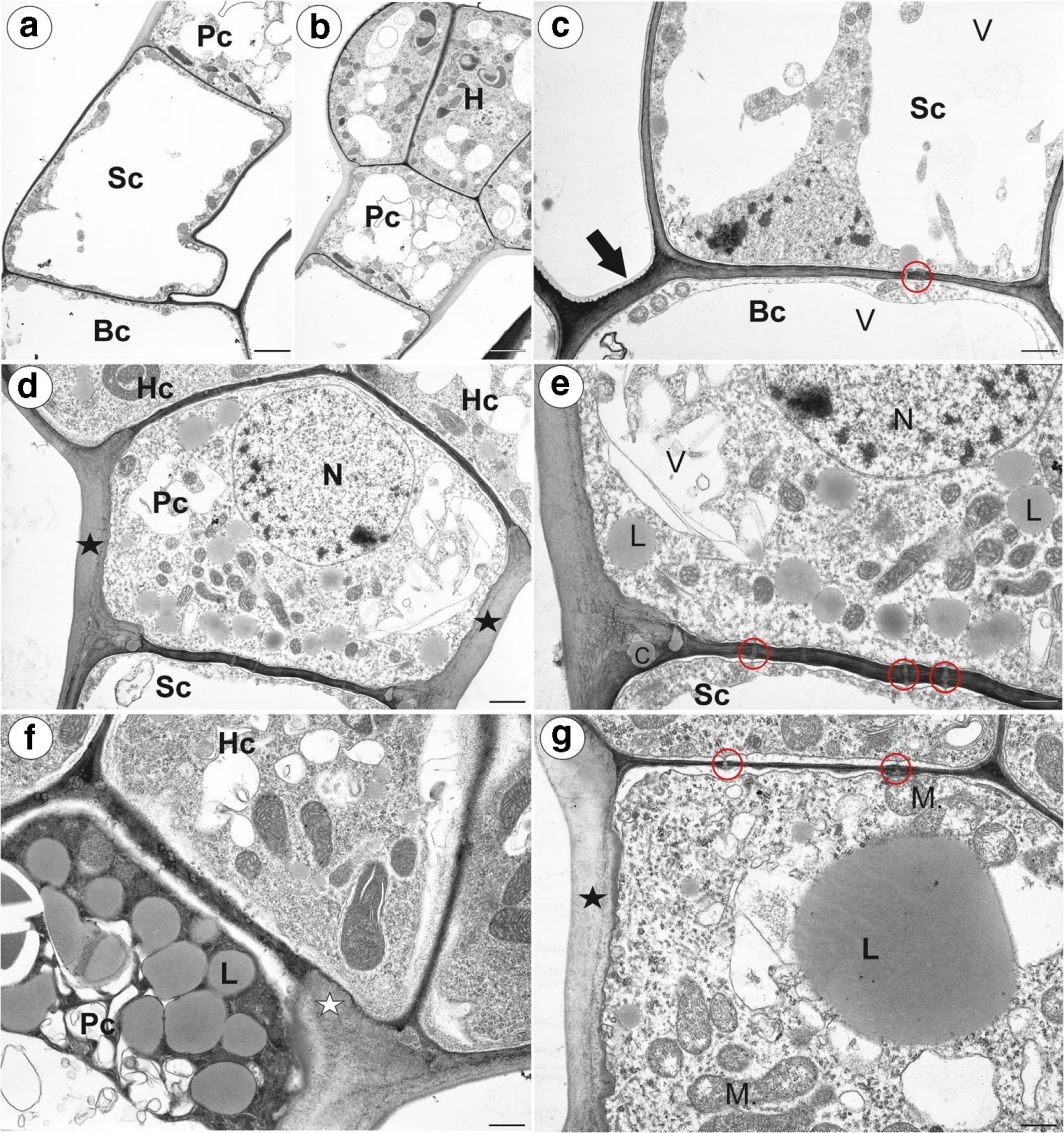
Ultrastructure of the nectary trichomes of *Pinguicula*; **a**, **b***P. emarginata* ultrastructure of whole nectary trichome: the basal cell (Bc), stalk cell (Sc), pedestal cell (Pc), head (H); scale bar = 2.7 μm and scale bar = 3.4 μm. **c***P. moctezumae* ultrastructure of the basal (Bc) and stalk cells (Sc): the cuticle of basal cell (arrow), plasmodesmata (red circle), vacuole (V); scale bar = 1.6 μm. **d–e***P. moctezumae* ultrastructure of the trichome cells; note that the lateral cell wall of the pedestal cell (Pc) was heavily impregnated with cutin (star): nucleus (N), vacuole (V), plasmodesmata (red circle), impregnation with cutin in a fragment of the transverse walls between the stalk cell and the pedestal cell (C), head cell (Hc), stalk cell (Sc); scale bar = 1.6 μm and scale bar = 0.8 μm. **f***P. moctezumae* accumulation of lipid bodies (L) in the pedestal cell: pedestal cell (Pc), head cell (Hc), impregnation with cutin in a fragment of the transverse walls between the pedestal cell and head cell (white star); scale bar = 0.8 μm. **g***P. moranensis.* Accumulation of lipid bodies (L) in the pedestal cell: mitochondria (M), plasmodesmata (red circle) between the pedestal cell and head cells, the lateral cell wall of the pedestal cell impregnated with cutin (star); scale bar = 0.9 μm

The trichomes of the nectary spur (Fig. 7a–o) were composed of a single basal cell, a stalk cell, a pedestal cell (barrier cell) and a multi-celled head (from four to eight cells) (Fig. 7c, e; Fig. 8a–c). In *P. moctezumae*, some trichomes were sessile and there was no stalk cell (Fig. 7a). Both the basal cell and stalk cell were highly vacuolated (Fig. 9a, b). The length and diameter of the trichome heads are listed in Table 1. Measurements of glandular trichomes length and trichomes head diameter revealed that the longest trichomes with widest trichomes head inside spur occurred in *P. esseriana* and *P. moranensis*. Analyses of trichomes length show the significant differences occurred in *P. esseriana* and *P. moranensis* comparing with *P. moctezumae*, *P. rectifolia* and *P. emarginata* (***p < 0.001) [supplementary Material]. We also observed marked differences in the trichomes head diameter between *P. esseriana* and *P. moranensis* comparing with *P. moctezumae* (**p < 0.01), *P. rectifolia* and *P. emarginata* (***p < 0.001) [supplementary Material]. *P. moctezumae* had wider trichomes head comparing with *P. emarginata* (**p < 0.01).

The outer wall of the basal cell developed a cuticle. The lateral wall of the stalk cell developed a distinct cuticle cell wall layer (Fig. 9c). The pedestal cell had a thick radial (lateral) wall, which was heavily impregnated with cutin (Fig. 9d). In the radial cell wall, two layers were distinguished (there were differences in the cell impregnation). The inner surface of the radial cell wall had an undulating surface (Fig. 9d, e and g). The impregnation with cutin also occurred in a fragment of the transverse walls between the stalk cell and the pedestal cell (Fig. 9e) as well as in the fragments of the cell walls between the pedestal cell and the head cells (Fig. 9f). However, the transverse walls were thinner than the radial cell wall and electron dense. Plasmodesmata occurred in the transverse walls between the stalk cell and the pedestal cell (Fig. 9e) as well as in the transverse walls between the pedestal cell and the terminal cells (Fig. 9g). The nucleus of the pedestal cell was prominent (Fig. 9d). The cytoplasm contained many mitochondria (Fig. 9d, e and g) and profiles of the rough endoplasmic reticulum. Plastids and microbodies were also observed. The vacuoles formed a reticulate network (Fig. 9e). One of the most prominent features of the pedestal cell was the presence of lipid bodies (Fig. 9e–g). In the pedestal cells, there was a large accumulation of lipid bodies that were almost equal in size (Fig. 9f), whereas in others, there was one large lipid body and a few very small ones (Fig. 9g). This, could be connected with fusing of lipid bodies.

**Fig. 10:**
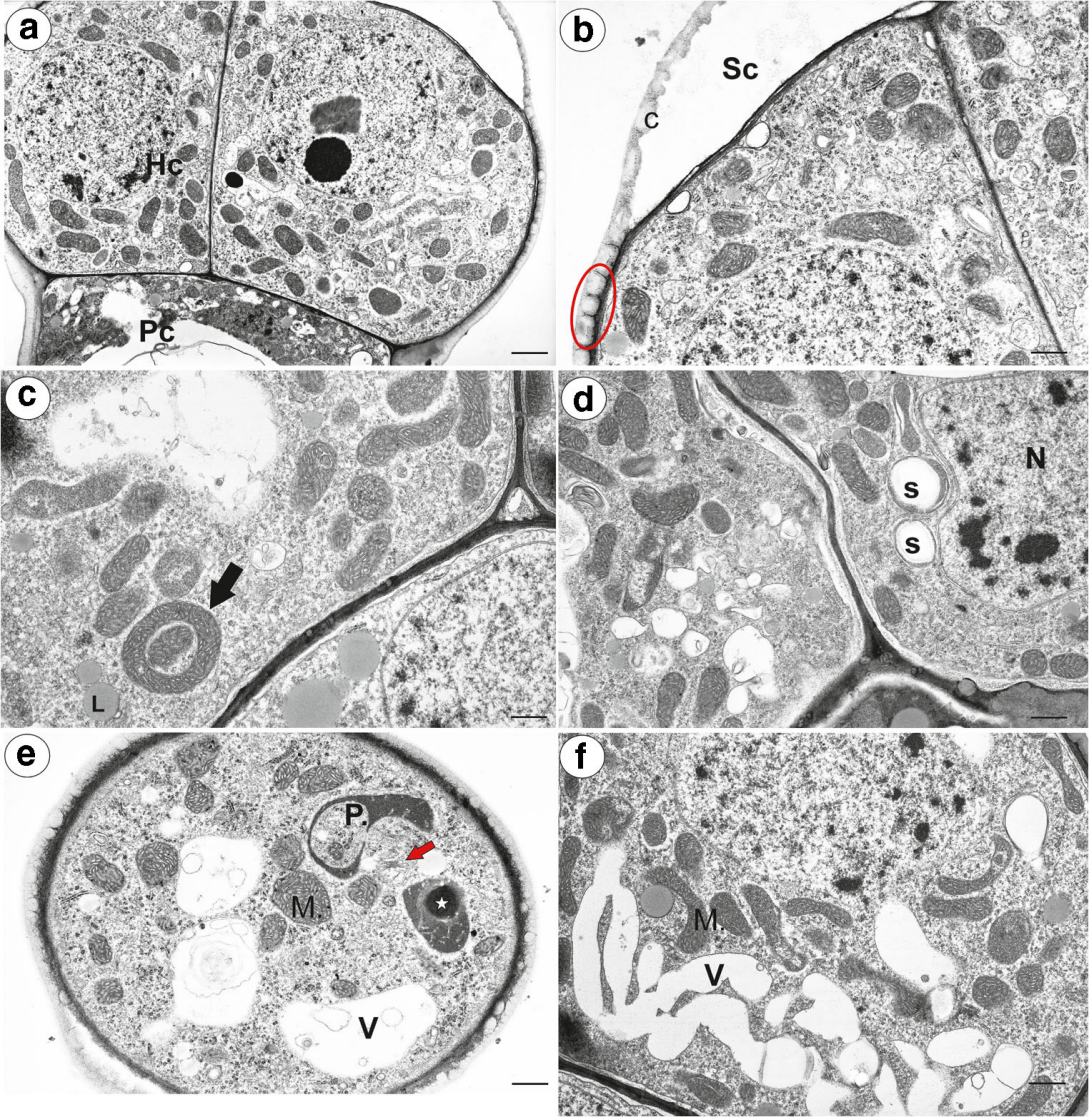
Ultrastructure of head cells of the *Pinguicula* nectary trichomes. **a**, **b** Ultrastructure of the head cells of *P. moranensis*: head cell (Hc), pedestal cell (Pc), subcuticular space (Sc), cuticle (C), polysaccharide fibrillary network in the cuticle layer (red eclipsed); scale bar = 1.7 μm and scale bar = 1 μm. **c**, **d** Ultrastructure of the head cells of *P. moctezumae*; note the numerous mitochondria some of which are cup-shaped (arrow), lipid droplets (L), plastids with starch (S), nucleus (N); scale bar =  1 μm and scale bar = 1.1 μm. **e** A part of a section through the head cell of *P. emarginata*; note the cup-shaped plastid, inclusion in plastid (star), mitochondria with well-developed cristae (M), small dictyosomes (red arrow), vacuole (V); scale bar = 1.1 μm. **f** A reticulate vacuole in the head cell of *P. moctezumae*: vacuole (V), mitochondria (M); scale bar = 0.8 μm

**Fig. 11:**
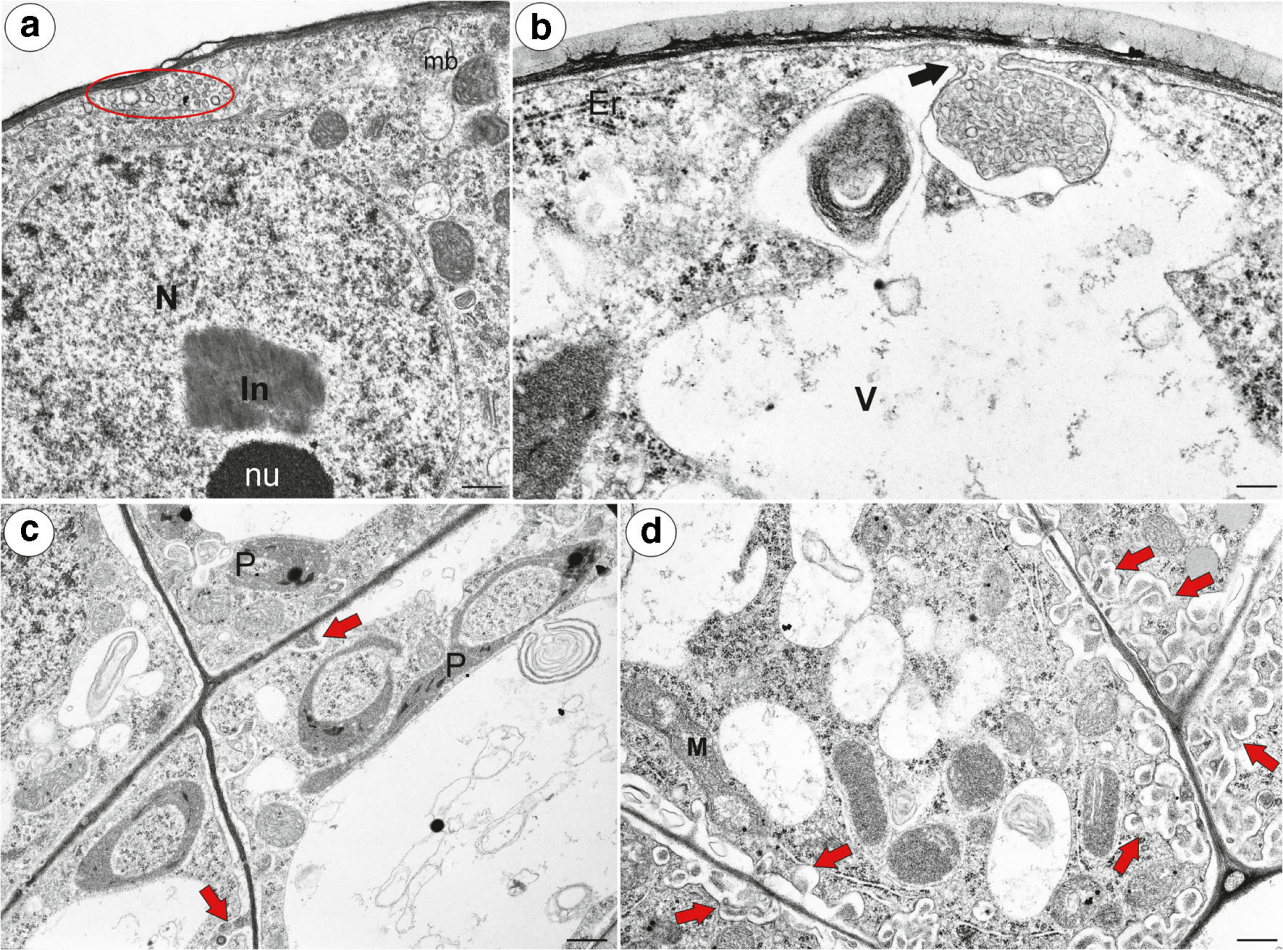
Ultrastructure of the head cells of the *Pinguicula* nectary trichomes. **a** Ultrastructure of the head cell of *P. moranensis*; note the large accumulation of membranous material in the periplasmic space (red eclipsed): nucleus (N), nucleolus (nu), paracrystalline protein inclusion (In); scale bar = 0.8 μm. **b** Ultrastructure of the head cell of *P. emarginata*; note the fusion of the large vesicle connected to the vacuole, which contains membranous material, with plasmalemma (arrow); endoplasmic reticulum (Er), vacuole (V); scale bar = 0.5 μm. **c** Ultrastructure of the head cells of *P. emarginata*; note the small cell wall ingrowths (red arrows); cup-shaped plastid (P); scale 1 bar = μm. **d** Ultrastructure of the head cells of *P. esseriana*; note the well-developed cell wall ingrowths (red arrows); scale bar = 0.9 μm

The head cells had a dense cytoplasm (Fig. 10a) with prominent nuclei with a paracrystalline protein inclusion (Fig. 10a and Fig. 11a). The cuticle became distended and separated from the cell walls and formed a subcuticular space at top part of the head (Fig. 10b). There was a polysaccharide fibrillary network of electron-dense ramification in the cuticle layer (Fig. 10b). Mitochondria were very numerous with well-developed cristae as view on the transversal section (Fig. 10c–f) and sometimes, they were cup-shaped (Fig. 10c). Plastids were numerous and were oval-, dumbbell- or cup-shaped as view on the transversal section (Fig. 10d, e and Fig. 11c). They had small starch grains (*P. moctezumae*, *P. moranensis*, *P. rectifolia*; (Fig. 10d)) and electron-dense inclusions (*P. emarginata*). The cytoplasm contained small lipid bodies, small dictyosomes (Fig. 10d–f) and microbodies (Fig. 11a). The vacuoles formed a reticulate network (Fig. 10f) or the vacuole was large. The vacuole contained some membranous material (Fig. 11c). In the periplasmic space (between the plasmalemma and the cell wall), there were vesicles. A large accumulation of vesicles and membranous material was sometimes observed in the periplasmic space (Fig. 11a). The fusion of a large vesicle that was connected to a vacuole that contained membranous material with plasmalemma was also observed (Fig. 11b). In *P. emarginata* and *P. esseriana*, the head cells were transfer cells; wall ingrowths occurred on the outer cell walls and on the transverse cell walls (Fig. 11c, d). In *P. esseriana*, the cell wall ingrowths were better developed (Fig. 11d) than in *P. emarginata* (Fig. 11c).

## Discussion

The results presented here show a conservative nectary trichome structure and spur anatomy in different *Pinguicula* species from Mexico. These similarities might stem from the fact that the examined species are closely related; thus, some traits may represent synapomorphies for the group. They are grouped into one clade—Mexican-Central American-Caribbean Clade I (Cieslak et al. 2005). From examined species, pollinators (butterflies) were recorded only for *P. moranensis* (Villegas and Alcalá 2018); however, flower structure (coloured flowers with long and thin spurs) suggests that also other examined species are pollinated by butterflies.

The gross structural similarities between the species that were examined were as follows: the spur anatomy, the occurrence of papillae, the architecture of the nectary trichomes and the ultrastructure characters of the trichome cells. However, there were some differences in the spur length (Fig. 1), the size of trichomes in the spurs, the occurrence starch grains in the spur parenchyma (they were not detected in *P. moranensis*) and the occurrence of cell wall ingrowths in the terminal cells of the nectary trichomes. In the nectaries, starch reserves are utilised as a source of carbohydrates to produce nectar (Nepi 2007); thus, the variations in the amount of starch among the examined *Pinguicula* species, as well as the lack of starch in the spurs of *P. moranensis*, might be caused by its hydrolysis.

We noted correlation between the length of the spur trichomes and the width of their heads. The species that had longest trichome stalk have also the largest diameter of trichome heads (*P. esseriana* and *P. moranensis*). It is interesting that the spur trichomes length and their head diameter were not correlated with the size of flowers (also spur length). *Pinguicula rectifolia* with large flowers had trichome sizes similar to *P. emarginata*, which had small flowers. We found a clear difference in the length of the spur trichomes as well as the width of their heads between *P. moranensis* and *P. rectifolia*. This characters may be an important diagnostic feature helpful in the determination of these species, especially that some authors included *P. rectifolia* in *P. moranensis* complex (e.g. Roccia et al. 2016); however, future analysis of more plant material from different populations is needed.

The spur anatomy of the *Pinguicula* that were examined here is very similar to *Utricularia* (Płachno et al. 2016, 2017, 2018, 2019b, c). In both genera, there are collateral vascular bundles in the nectaries (spurs). Papillose surfaces of the internal spur epidermis occur in the spurs of all Lentibulariaceae genera (*Utricularia*—Clivati et al. 2014; Płachno et al. 2016, 2017, 2018; *Genlisea*—Aranguren et al. 2018). However, there is variability in the case of the occurrence of cuticular striations among the species.

Stomata are described on the surfaces of the internal spur epidermis for Lentibulariaceae for first time. In many plants, nectaries with modified stomata (‘nectarostomata’) occur, which are used to release nectar (e.g. Davis and Gunning 1992; Davies et al. 2005; Wist and Davis 2006; Nepi 2007; Pacini and Nepi 2007). In some species, nectar is secreted by both trichomes and modified stomata in spur in the same plant (e.g. in *Tropaeolun majus*; Rachmilevitz and Fahn 1975). However, there are glandular parenchyma in these nectaries that produce nectar. In the examined flowers of *Pinguicula*, the spur parenchyma cells are not glandular; thus, the stomata probably do not participate in the release of nectar. Explaining their function requires further research.

Similar nectary capitate trichomes, as are described here, have been recorded in the spurs of various *Utricularia* species (Farooq 1963; Farooq and Siddiqui 1966; Clivati et al. 2014; Płachno et al. 2016, 2017, 2018, 2019b, c) and *Genlisea violacea* (Aranguren et al. 2018). Unfortunately, there are no published data about the ultrastructure of the nectary trichomes in *Genlisea*. The ultrastructural similarities between the nectary trichomes in *Pinguicula* and *Utricularia* are as follows: a highly vacuolated basal cell, and sometimes, also a stalk cell; a pedestal with impregnated with cutin radial wall (Casparian strip); in the terminal cells: the dense cytoplasm contains large amount of organelles—numerous mitochondria and multi-shaped plastids (which evidence a highly metabolic function); a paracrystalline protein inclusion in the nucleus and the occurrence of cell wall ingrowths, a thick cuticle and a subcuticular space for accumulating nectar. The ultrastructure characters of the *Pinguicula* nectary trichome terminal cells are typical for nectaries (see Nepi 2007; Pacini and Nepi 2007).

In the cuticular layer of head cells in the examined *Pinguicula*, there were polysaccharide micro-canals, which are hydrophilic pathways that most probably form pathways for the release of secretions (Paiva 2016, 2017). Such a structure has been described in the cells of various nectary types in many species (e.g. Stpiczyńska 2003; Wist and Davis 2006; Rocha and Machado 2009; Antoń and Kamińska 2015; Weryszko-Chmielewska and Chwil 2016) as well as in other plant glandular structures—osmophores (García et al. 2007; Płachno et al. 2010; Kowalkowska et al. 2012, 2014, 2017; Paiva et al. 2019), collectors (Tresmondi et al. 2017) and trichomes, which produce a lipophilic secretion (Machado et al. 2017; Muravnik et al. 2019). Interestingly, we found polysaccharide micro-canals in the cuticular layer of the spur papillae. These structures were recorded in the spur papillae of *Utricularia* (Płachno et al. 2016). However, in both *Pinguicula* and *Utricularia*, the papillae in the mature spur are not glandular. Therefore, we speculate that the occurrence of these micro-canals may indicate that the papillae participate in the reabsorption of nectar.

We observed lipid droplets in the cytoplasm in the nectary trichome cells. Lipid droplets have previously been recorded in the cytoplasm in the nectary trichome cells of *Utricularia multifida* (Płachno et al. 2019c). Machado et al. (2017) proposed that the occurrence of lipid droplets in the cytoplasm in the nectary cells might indicate that the nectar is enriched with lipids. However, we observed a large accumulation of lipid droplets in the pedestal cells, which play the role of barrier cells. A similar accumulation of lipids was recorded in the pedestal cells of trichomes of *Utricularia* turions (Płachno et al. 2014), and therefore, the occurrence of lipid droplets in the pedestal cells might not be directly connected with the secretion of nectar.

Płachno et al. (2018, 2019c) proposed that the nectar secretion in *Utricularia* occurs via an eccrine mode. This type of nectar secretion probably occurs in *P. esseriana* due to its well-developed cell wall ingrowths. However, in other *Pinguicula* species (*P. moctezumae*, *P. moranensis*) that have been examined, there are no cell wall ingrowths in the nectary glandular cells or, if they are present, they are very weakly developed (*P. emarginata*). In these species, we observed material in the periplasmic space as well as the fusion of a large vesicle that was connected to the vacuole that contained membranous material with the plasmalemma (Fig. 11b). Paiva (2016) proposed cyclic mechanical actions of the protoplast in the secretory cells, which “in the form of successive cycles of contraction and expansion, causes the material accumulated in the periplasmic space to cross the cell wall and the cuticle” (Pavia 2016, pg. 533). He proposed that during the fusion of the vacuolar and plasma membranes, the vacuolar content is released into the periplasmic space. Thus, we speculate that in some *Pinguicula* species, the secretion of nectar may occur via a mode other than the eccrine mode. However, this problem requires further research, including studies of the various stages of the development of the nectary *Pinguicula* trichomes.

To conclude, we show that both the nectary trichome structure and the spur anatomy in different *Pinguicula* species from Mexico are conservative. Similar nectary capitate trichomes, as are described here, have been recorded in the spurs of species from other Lentibulariaceae genera. There are many ultrastructural similarities between the nectary trichomes in *Pinguicula* and *Utricularia*.

## Electronic supplementary material

ESM 1 Trichomes length (mean ± SD) for each examined *Pinguicula* species. Significant differences in the length of trichomes between particular species are denoted as ***p < 0.001. (PNG 50 kb)

High Resolution Image (TIF 5910 kb)

ESM 2 Trichomes head diameter (mean ± SD) for each examined *Pinguicula* species. Significant differences in the head diameter of trichomes between particular species are denoted as **p < 0.01, and ***p < 0.001. (PNG 59 kb)

High Resolution Image (TIF 6029 kb)
