# Supplementary figures and images for: Flower nectar trichome structure of carnivorous plants from the genus butterworts Pinguicula L. (Lentibulariaceae)
Source: Protoplasma. 2019 Aug 19;257(1):245–59. doi: 10.1007/s00709-019-01433-8 (PMC6982637; doi:10.1007/s00709-019-01433-8)

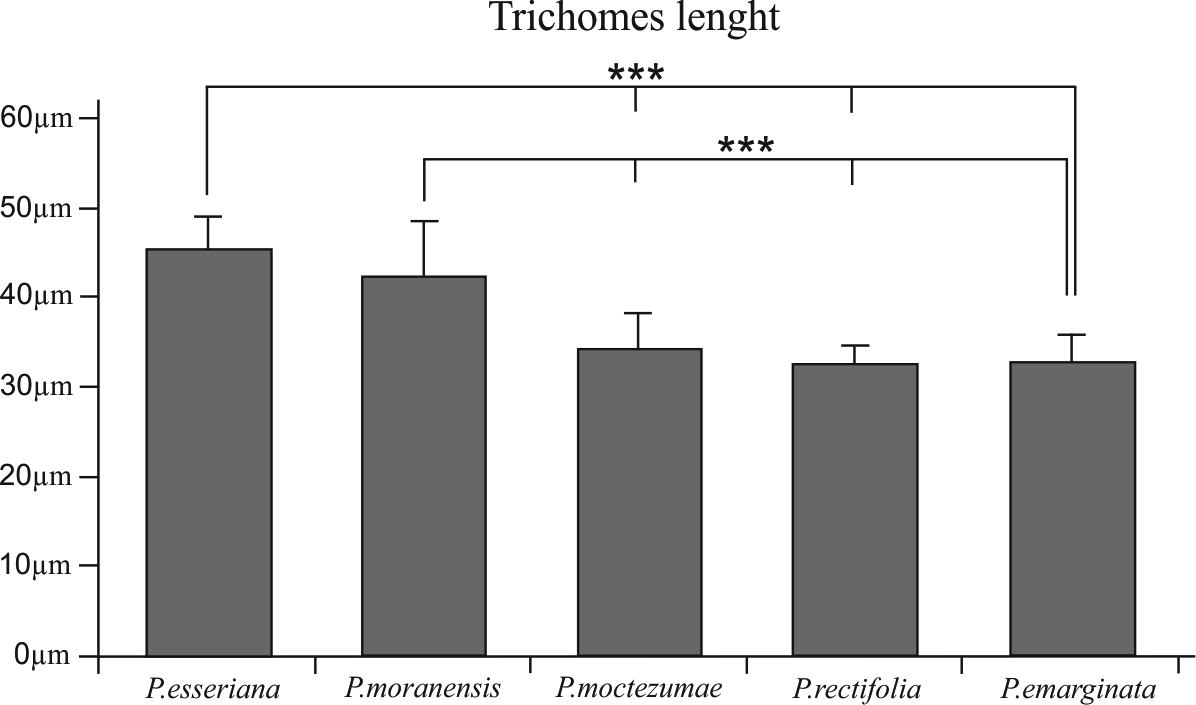

Supplement: Supplementary file 1 — Trichomes length (mean ± SD) for each examined Pinguicula species. Significant differences in the length of trichomes between particular species are denoted as ***p < 0.001. (PNG 50 kb) [file 709_2019_1433_Fig12_ESM.png]

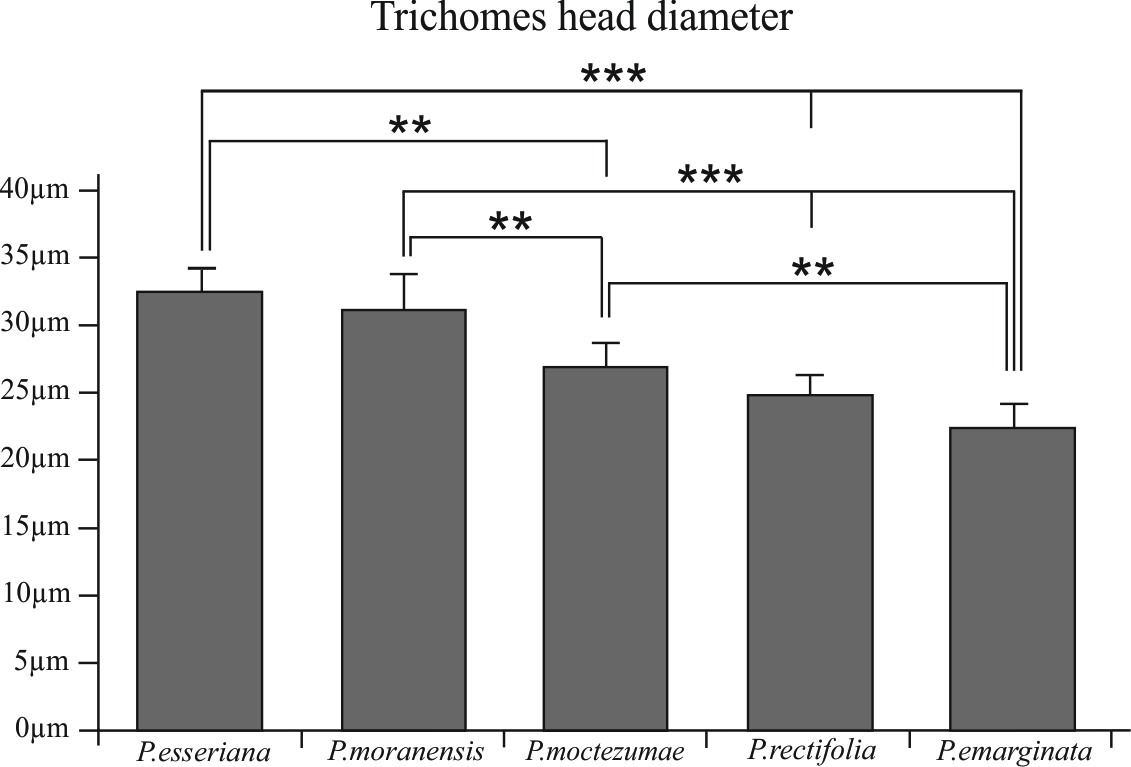

Supplement: Supplementary file 3 — Trichomes head diameter (mean ± SD) for each examined Pinguicula species. Significant differences in the head diameter of trichomes between particular species are denoted as **p < 0.01, and ***p < 0.001. (PNG 59 kb) [file 709_2019_1433_Fig13_ESM.png]
